# Excellent Magnetocaloric Performance of the Fe_87_Ce_13−x_B_x_ (x = 5, 6, 7) Metallic Glasses and Their Composite

**DOI:** 10.3390/ma16124393

**Published:** 2023-06-14

**Authors:** Shu-Hui Zheng, Qiang Wang, Li-Ze Zhu, Peng-Jie Wang, Ding Ding, Ben-Zhen Tang, Peng Yu, Jin-Lei Yao, Lei Xia

**Affiliations:** 1 Institute of Materials & Laboratory for Microstructure, Shanghai University, Shanghai 200072, China; zhengshuhui@shu.edu.cn (S.-H.Z.); mat_wq@shu.edu.cn (Q.W.); lize_zhu@163.com (L.-Z.Z.); w_pengjie@163.com (P.-J.W.); d.ding@shu.edu.cn (D.D.); 2 Chongqing Key Laboratory of Photo-Electric Functional Materials, College of Physics and Electronic Engineering, Chongqing Normal University, Chongqing 401331, China; bz.tang@cqnu.edu.cn (B.-Z.T.); pengyu@cqnu.edu.cn (P.Y.); 3 Jiangsu Key Laboratory of Micro and Nano Heat Fluid Flow Technology and Energy Application, School of Physical Science and Technology, Suzhou University of Science and Technology, Suzhou 215009, China; jlyao@usts.edu.cn

**Keywords:** metallic glass, glass-forming ability, Curie temperature, table-like magnetic entropy change profile

## Abstract

The novel Fe_87_Ce_13−x_B_x_ (x = 5, 6, 7) metallic glass (MG) ribbons were fabricated in this work. The compositional dependence of glass forming ability (GFA), magnetic and magnetocaloric properties of these ternary MGs, and the mechanism involved was investigated. The GFA and Curie temperature (*T_c_*) of the MG ribbons were found to improve with the boron content, and the peak value of magnetic entropy change (−Δ*S_m_^peak^*) reaches a maximum of 3.88 J/(kg × K) under 5 T when x = 6. Based on the three results, we designed an amorphous composite that exhibits a table-shape magnetic entropy change (−Δ*S_m_*) profile with a rather high average −Δ*S_m_* (−Δ*S_m_^average^*~3.29 J/(kg × K) under 5 T) from 282.5 K to 320 K, which makes it a potential candidate for the highly efficient refrigerant in a domestic magnetic refrigeration appliance.

## 1. Introduction

Recently, the demand for refrigeration has risen sharply because of the ongoing and extremely high-temperature weather. However, conventional refrigeration technology can make this problem worse due to the generation of ozone-depleting gases by refrigerants. A novel refrigeration technology, namely magnetic refrigeration (MR, which has the advantages of high efficiency, environmental friendliness, great stability and reliability, low noise, and easy miniaturization [1,2,3,4,5]), may be a better choice. The MR technology was first used by Gauque in 1933 to achieve ultra-low temperatures below 1 K and has been applied to the low and medium temperature regions up to now [6,7], although it is still in the research stage at high temperatures, especially for civil applications at room temperature. One of the key issues affecting the civil application of MR is the magnetocaloric effect (MCE) performance of refrigerants.

Although a large number of compounds with ultrahigh magnetic entropy change (−Δ*S_m_*) have been developed, their narrow working temperature ranges make them difficult to synthesize a composite material with a flattened −Δ*S_m_* curve [8,9,10,11], which is of great significance for the optimization of cooling efficiency in the Ericsson cycle [12]. Additionally, the presence of magnetic hysteresis and thermal hysteresis in these compounds prevents them from operating at high frequencies with fast response. On the contrary, the metallic glasses (MGs) that exhibit a broader −Δ*S_m_* peak (−Δ*S_m_^peak^*) without magnetic hysteresis and thermal hysteresis are preferred, such as Gd-based and Fe-based MGs [13,14,15,16,17,18,19,20,21,22,23,24,25,26]. The tunable Curie temperature (*T_c_*) in a wide range of composition adjustments is beneficial for constructing the flattened −Δ*S_m_* curve [15,16,17]. Furthermore, the advantages of outstanding mechanical, physical, and chemical properties can also ensure that MGs have a longer service life and numerous application scenarios [27,28]. For example, Zr-based or Ti-based MGs with both ultra-high strength and plasticity have made significant progress in key structural materials for military and aerospace equipment; Ca-based, Mg-based, or Fe-based MGs possess good biocompatibility and comprehensive mechanical properties and are expected to be used in artificial bones.

Compared with rare-earth (RE)-based MGs, Fe-based MGs show greater application value in civil aspects (such as magnetic refrigerators, air conditioners, wine cabinets, and so on) due to their lower cost and better glass forming ability (GFA), but the −Δ*S_m_^peak^* of these Fe-based MGs is not as high as that of RE-based MGs [15,16,17,18,19,20,21,22,23,24,25,26]. Therefore, improving the −Δ*S_m_^peak^* of the Fe-based MGs as much as possible is a top priority for the civil application of MR technology. Amongst these reported Fe-based MGs, Fe-Zr-B-based MG ribbons are the outstanding ones [15,16,17,18,23,24,25,26]. For example, the Fe-Zr-B ternary amorphous ribbons exhibited a −Δ*S_m_^peak^* under 5 T of 2.75–3.34 J/(kg × K) at the *T_c_* ranging from 271 K to 327 K [18,24]. By minor element substitution, the −Δ*S_m_^peak^* under 5 T of the multicomponent Fe-Zr-B-based MG ribbons was increased to a maximum value of 3.55 J/(kg × K) at 336 K [26]. Unfortunately, the values of −Δ*S_m_^peak^* in these Fe-Zr-B-based MG ribbons are still not as high for the practical application of MR. In our recent works, we found that Fe-La/Ce-B-based glassy ribbons show rather excellent MCE near room temperature, and the −Δ*S_m_^peak^* under 5 T of Fe_87_Ce_8_B_5_ ribbon reaches 3.65 J/(kg × K) at 283 K, which is 12.3% higher than that of Fe_87_Zr_8_B_5_ metallic glass and exceeds the −Δ*S_m_^peak^* of most other Fe-Zr-B-based amorphous alloys [29,30]. The large −Δ*S_m_^peak^* of the Fe_87_Ce_8_B_5_ amorphous ribbon was attributed to the extra magnetic moment of the Ce atom, but the effect of Ce content on the −Δ*S_m_^peak^* of the Fe-Ce-B MG ribbons and the involved mechanism is still unclear. The detailed investigation of the magnetic and magnetocaloric properties of the Fe-Ce-B ternary MG ribbons will be helpful for the clarification of the effects of the Ce content on the magnetic properties and MCE of the Fe-Ce-B MG ribbons and the further development of the multicomponent Fe-based amorphous alloys with better MCE near room temperature. As such, we prepared the Fe_87_Ce_13−x_B_x_ (x = 5, 6, 7) amorphous ribbons using melt-spinning technology and systematically investigated the influences of boron on GFA, *T_c_* and −Δ*S_m_^peak^* of the three ribbons, as well as the mechanisms involved, in this paper. Finally, an amorphous composite with a flattened −Δ*S_m_* profile was designed according to the three samples.

## 2. Materials and Methods

The Fe_87_Ce_13−x_B_x_ (x = 5, 6, 7) alloy ingots were prepared by melting high-purity raw materials (≥99.9 at%) using a vacuum arc-melting furnace with the protection of Ar gas. Each ingot of the Fe_87_Ce_13−x_B_x_ (x = 5, 6, 7) master alloys was melted more than four times to ensure their compositional homogeneity. These ingots were remelted in a quartz tube again and sprayed onto the surface of a high-speed (~55 m/s) rotating copper roller to form the Fe_87_Ce_13−x_B_x_ (x = 5, 6, 7) ribbons. The structure of these ribbons was characterized by a PANalytical X-ray diffractometer with Cu *K_α_* radiation. The differential scanning calorimetry (DSC) traces were measured at a heating rate of 20 K/min on a Netzsch DSC 404C calorimeter to evaluate the GFA of the Fe_87_Ce_13−x_B_x_ (x = 5, 6, 7) ribbons. The magnetic measurements of the samples, including hysteresis loops, magnetization vs. temperature (*M*-*T*) curves, and isothermal magnetization (*M*-*H*) curves, were performed on a vibrating sample magnetometer (VSM), which is a module of 6000 Physical Property Measurement System (PPMS, Quantum Design).

## 3. Results and Discussion

**Figure 1:**
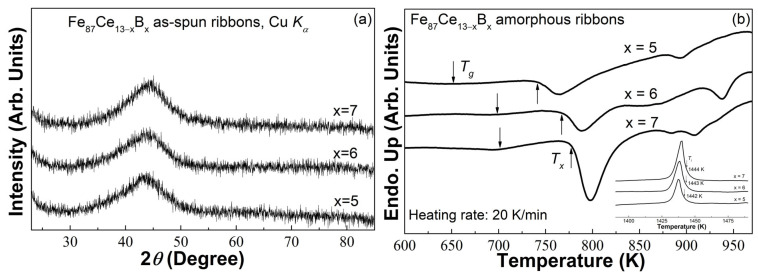
(**a**) The XRD patterns of the Fe_87_Ce_13−x_B_x_ (x = 5, 6, 7) as-spun ribbons; (**b**) the DSC traces of the Fe_87_Ce_13−x_B_x_ (x = 5, 6, 7) amorphous ribbons at the heating rate of 20 K/min; the inset is the melting behaviors.

Figure 1a shows the XRD patterns of the Fe_87_Ce_13−x_B_x_ (x = 5, 6, 7) ribbons. Only smooth diffraction humps between 40° and 50° and no obvious crystalline peaks were observed in these patterns, indicating the amorphous structure of the Fe_87_Ce_13−x_B_x_ (x = 5, 6, 7) ribbons. Figure 1b displays the DSC curves of the Fe_87_Ce_13−x_B_x_ (x = 5, 6, 7) amorphous ribbons. A weak glass transition endothermic hump before a sharp crystallization exothermic peak was found in each DSC curve, which further confirms the glassy characteristics of the three ribbons. From their DSC curves, we can obtain the thermal parameters, including the onset temperature of glass transition (*T_g_^onset^*) and crystallization (*T_x_^onset^*), and liquidus temperature (*T_l_*), of the Fe_87_Ce_13−x_B_x_ (x = 5, 6, 7) MG ribbons, as listed in Table 1. As a result, the reduced glass transition temperature (*T_rg_* = *T_g_/T_l_*) [31] and the parameter *γ* (=*T_g_*/(*T_g_* + *T_l_*)) [32] can be calculated accordingly to estimate the GFA of these ribbons. The *T_rg_* and *γ* are about 0.451 and 0.354 for x = 5, 0.483 and 0.358 for x = 6, and 0.485 and 0.362 for x = 7, respectively. Both the *T_rg_* and *γ* increase with the increase of boron content, which indicates that the addition of boron can improve the GFA of the Fe_87_Ce_13−x_B_x_ (x = 5, 6, 7) MG ribbons.

**Figure 2:**
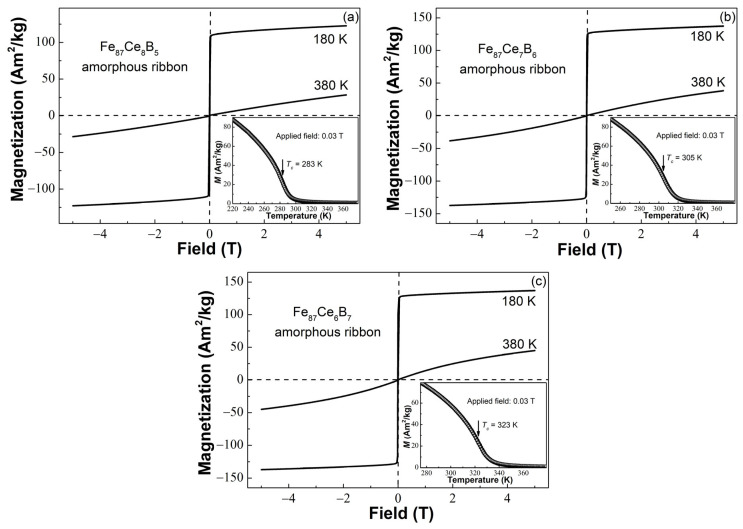
The hysteresis loops of the (**a**) Fe_87_Ce_8_B_5_, (**b**) Fe_87_Ce_7_B_6_, and (**c**) Fe_87_Ce_6_B_7_ amorphous ribbons measured at 180 K and 380 K under 5 T; the insets are their *M*-*T* curves measured under 0.03 T.

The hysteresis loops at 180 K and 380 K of the Fe_87_Ce_13−x_B_x_ (x = 5, 6, 7) glassy ribbons were measured under the maximum field of 5 T, as shown in Figure 2a–c. All glassy ribbons are paramagnetic at 380 K and soft magnetic with near zero hystereses at 180 K, which suggests that these ribbons transform from ferromagnetism to paramagnetism between 180 K and 380 K. The saturation magnetizations (*M_s_*) at 180 K of the three samples are about 129.4 Am^2^/kg, 143.2 Am^2^/kg, and 142.1 Am^2^/kg, respectively. The *M_s_* first increases and then slightly decreases with the boron content. To determine the ferromagnetic–paramagnetic transition temperature of the Fe_87_Ce_13−x_B_x_ (x = 5, 6, 7) MG ribbons, we measured their *M*-*T* curves from 180 K to 380 K under 0.03 T after cooling these samples without any applied fields. As illustrated in the insets of Figure 2a–c, all *M*-*T* curves of the Fe_87_Ce_13−x_B_x_ (x = 5, 6, 7) ribbons show similar shapes, and *T_c_* can be deduced to be ~283 K for x = 5, ~305 K for x = 6, and ~323 K for x = 7, respectively. It was found that *T_c_* of the Fe_87_Ce_13−x_B_x_ (x = 5, 6, 7) ribbons almost linearly increases with the increase of boron content. The Curie temperature of the Fe_87_Ce_13−x_B_x_ (x = 5, 6, 7) glassy ribbons should also be decided by the interaction between Fe atoms because of only one electron in the 4*f* shell of the Ce atom. The monotonically increasing relationship between *T_c_* and composition was also observed in the Fe-Zr-B ternary amorphous ribbons, which is believed to be due to the enhanced 3*d*–3*d* direct interaction between Fe atoms by the addition of boron [18,24]. In addition, the increase of boron content (namely the reduction of Ce content) will diminish the antiferromagnetic coupling between Ce and Fe atoms, which can dilute the direct interaction between Fe atoms [30] and, thus, result in the enhancement of 3*d*–3*d* interaction between Fe atoms.

**Figure 3:**
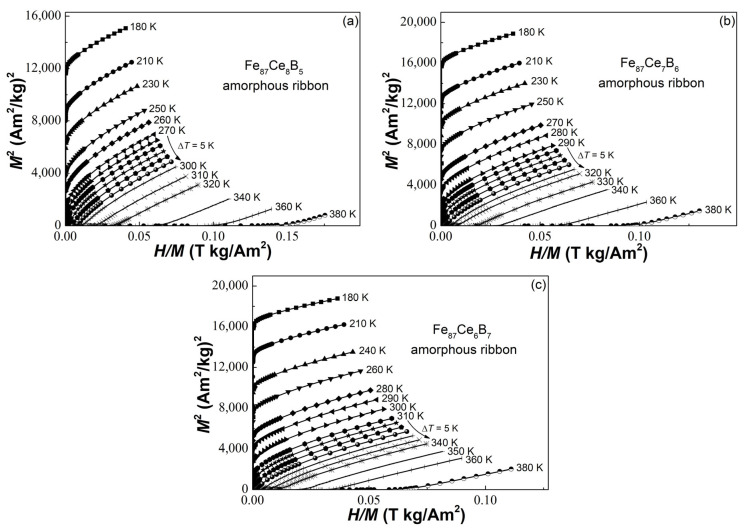
The Arrott plots of the (**a**) Fe_87_Ce_8_B_5_, (**b**) Fe_87_Ce_7_B_6_, and (**c**) Fe_87_Ce_6_B_7_ amorphous ribbons.

The Arrott plots can be deduced from their isothermal magnetization curves (not present in the paper) to judge the types of magnetic transition in the Fe_87_Ce_13−x_B_x_ (x = 5, 6, 7) amorphous ribbons, as illustrated in Figure 3a–c. The slope of each Arrott plot at every temperature is positive, without “S” or “C”-shaped feature, indicating that both the three alloys experience a second-order ferromagnetic–paramagnetic transition near their *T_c_* [33].

In order to characterize MCE of the second-order phase transition Fe_87_Ce_13−x_B_x_ (x = 5, 6, 7) amorphous ribbons, the magnetic entropy change can be calculated by Maxwell equation [34]:

(1) $$\Delta S_{m}\left(T,H\right){=S}_{m}\left(T,H\right)-S_{m}\left(T,0\right)=\int_{0}^{H}{\left(\frac{\partial M}{\partial T}\right)}_{H}\mathrm{dH}$$

**Figure 4:**
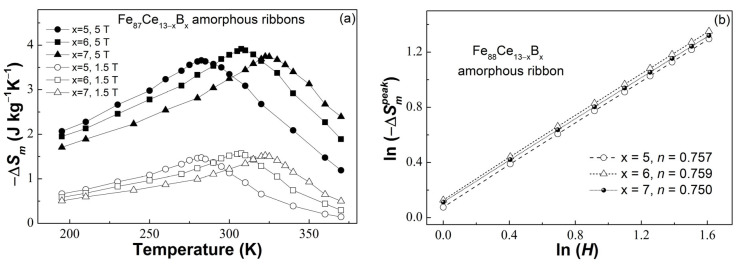
(**a**) The (−Δ*S_m_*)-*T* curves of the Fe_87_Ce_13−x_B_x_ (x = 5, 6, 7) amorphous ribbons under 1.5 T and 5 T; (**b**) the ln(−Δ*S_m_^peak^*) vs. ln(*H*) plots and their linear fittings of the Fe_87_Ce_13−x_B_x_ (x = 5, 6, 7) amorphous ribbons.

Figure 4a shows the temperature dependence of −∆*S_m_* ((−∆*S_m_*)-*T* curves) for the Fe_87_Ce_13−x_B_x_ (x = 5, 6, 7) ribbons under the magnetic fields of 1.5 T and 5 T. The broadened distribution of each (−∆*S_m_*)-*T* curve suggests the typical feature of second-order magnetic transition materials. The −Δ*S_m_^peak^* under 1.5 T and 5 T of the Fe_87_Ce_13−x_B_x_ (x = 5, 6, 7) ribbons are summarized in Table 1. We can find that the value of −Δ*S_m_^peak^* first increases and then decreases with the increase of boron content and reaches a maximum value of ~3.88 J/(kg × K) under 5 T at x = 6. The variation is the same as that in *M_s_*. Although the B substitution for Ce can improve the 3*d*–3*d* interaction between Fe atoms, which is beneficial for the enhancement of −Δ*S_m_^peak^*, the decrease of the Ce atom simultaneously reduces the total magnetic entropy of the alloy, thus resulting in the reduction of −Δ*S_m_^peak^*, as the Ce atom also contributes to the magnetic entropy change [18,24,30]. It may be precisely the two opposing factors that lead to this variation in *M_s_* and −Δ*S_m_^peak^*. According to the −Δ*S_m_^peak^* under various magnetic fields, the field dependence of −Δ*S_m_^peak^* for the Fe_87_Ce_13−x_B_x_ (x = 5, 6, 7) ribbons can be investigated by constructing the ln(−Δ*S_m_^peak^*)-ln(*H*) plots, as shown in Figure 4b. Each set of point plots is linearly fitted well, and the slope (expressed by *n*) of the linear fitting is 0.756 for x = 5, 0.759 for x = 6, and 0.750 for x = 7, respectively. The values of *n* are consistent with that of other MGs [16,17,18,23,24,25,26,29,30] but slightly larger than the predicted value of the mean field model [35]. This deviation is mainly caused by local inhomogeneity in MGs because the presence of short-range ordered clusters in MGs leads to a magnetic transformation in a wide temperature range [17].

**Figure 5:**
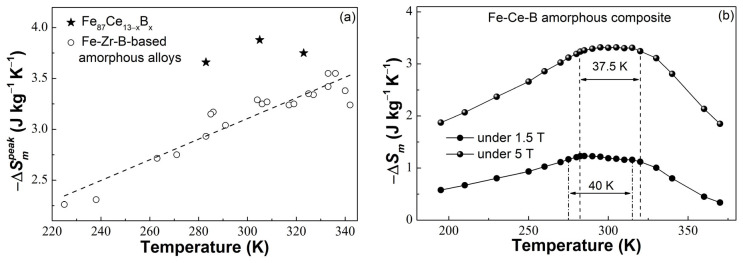
(**a**) The −Δ*S_m_^peak^* of the Fe_87_Ce_13−x_B_x_ (x = 5, 6, 7) amorphous ribbons and some Fe-Zr-B-based amorphous alloys under 5 T; (**b**) the flattened (−∆*S_m_*)-*T* curves of the amorphous composite composed of the three amorphous ribbons under 1.5 T and 5 T.

Figure 5a displays the −Δ*S_m_^peak^* under 5 T of the Fe_87_Ce_13−x_B_x_ (x = 5, 6, 7) amorphous ribbons and some Fe-Zr-B-based MGs [15,16,17,18,23,24,25,26,36]. A positive correlation between −Δ*S_m_^peak^* and *T_c_* was observed in the Fe-Zr-B-based MGs, implying that the higher −Δ*S_m_^peak^* can only be achieved at higher temperatures for the Fe-Zr-B-based MGs, which is very unfriendly for the application of magnetic refrigeration at room temperature. However, the novel Fe_87_Ce_13−x_B_x_ (x = 5, 6, 7) amorphous ribbons prepared in this work provide a better idea and exhibit better magnetocaloric performance than the Fe-Zr-B-based MGs. For example, the lowest −Δ*S_m_^peak^* in these amorphous ribbons (~3.66 J/(kg × K) at 282.5 K for Fe_87_Ce_8_B_5_) is still 3.1% higher than the largest −Δ*S_m_^peak^* in the Fe-Zr-B-based MGs (~3.55 J/(kg × K), while *T_c_* reaches around 335 K [15,25]). The Fe_87_Ce_7_B_6_ ribbon with the largest −Δ*S_m_^peak^* shows a value at least 9.3% higher than that of the Fe-Zr-B-based MGs. Additionally, the −Δ*S_m_^peak^* of the Fe_87_Ce_13−x_B_x_ (x = 5, 6, 7) amorphous ribbons is much higher than that of the Fe-Zr-B-based MGs with the same *T_c_*, which can be intuitively observed from Figure 5a.

On the other hand, the *T_c_* of the Fe_87_Ce_13−x_B_x_ (x = 5, 6, 7) amorphous ribbons increases from 283 K to 323 K, which perfectly matches the temperature interval of a domestic magnetic refrigeration appliance. Therefore, we can construct an amorphous composite with a flattened −∆*S_m_* profile that is the requirement of application in an Ericsson cycle according to the three ribbons [37]. The −∆*S_m_* of the composite can be calculated as follows:

(2) $$-\Delta S_{m}\left(\mathrm{composite}\right)=\sum_{i=1,2,\ldots ,n}^{n}\;w_{i}\;\times \;(-\Delta S_{m}{)}_{i}$$

where *w_i_* is the weight fraction of each component; the validity of Equation (2) has been verified in Ref. [38]. Therefore, by adjusting the weight fraction of these ribbons according to Equation (2), the amorphous composite was designed to be synthesized with 44% (wt.%) Fe_87_Ce_8_B_5_ + 3% (wt.%) Fe_87_Ce_7_B_6_ + 53% (wt.%) Fe_87_Ce_6_B_7_ amorphous ribbons. As shown in Figure 5b, the −∆*S_m_* of the amorphous composite fluctuates with an average value of ~1.20 J/(kg × K) from 275 K to 315 K (∆*T_table_* = 40 K) under 1.5 T, and ~3.29 J/(kg × K) from 282.5 K to 320 K (∆*T_table_* = 37.5 K) under 5 T. Compared with the Fe-Zr-B-based amorphous composite materials, an amorphous hybrid with a larger average −∆*S_m_* (−∆*S_m_^average^*) was achieved in this work [15,16,17]. In addition, the effective cooling capacity (*RC_eff_* = −∆*S_m_^average^* × ∆*T_table_* [39]) used to assess the actual cooling capacity of the Fe-Ce-B amorphous hybrid was calculated to be about 48.0 J/kg under 1.5 T and 123.4 J/kg under 5 T, both of which are also higher than those of the Fe-Zr-B-based amorphous composites with a similar ∆*T_table_* [15,17,39]. Furthermore, considering that the components of the amorphous composite do not contain radioactive elements, the Fe-Ce-B amorphous hybrid, thereby, shows a better application potential as highly efficient refrigerants in a domestic magnetic refrigeration appliance.

## 4. Conclusions

In summary, we successfully prepared the Fe_87_Ce_13−x_B_x_ (x = 5, 6, 7) amorphous ribbons, and the influences of boron addition on their GFA, *T_c,_* and −Δ*S_m_^peak^* were systematically investigated. The main conclusions are as follows:

(i) The *T_rg_* and *γ* indicate that the GFA of the Fe_87_Ce_13−x_B_x_ (x = 5, 6, 7) ribbons were increased with the addition of boron;
(ii) All ribbons show soft magnetic properties with negligible coercivity at 180 K, and *T_c_* of the Fe_87_Ce_13−x_B_x_ (x = 5, 6, 7) amorphous ribbons is ~283 K for x = 5, ~305 K for x = 6, and ~323 K for x = 7, respectively. The increased *T_c_* with the addition of boron may be due to the enhanced 3*d*–3*d* interaction between Fe atoms induced by the increase of boron content and the reduction of Ce content;
(iii) The −Δ*S_m_^peak^* of the Fe_87_Ce_13−x_B_x_ (x = 5, 6, 7) amorphous ribbons first increases and subsequently decreases with the increase of boron content, which is supposed to be related to the competitive effect between the enhanced 3*d*–3*d* interaction by the addition of B content and the reduced total magnetic entropy by the decrease of Ce content;
(iv) Both Arrott plots and the value of *n* at *T_c_* suggest the typical second-order magnetic transition magnetocaloric behavior of the three ribbons. All the Fe_87_Ce_13−x_B_x_ (x = 5, 6, 7) amorphous ribbons show rather excellent MCE than Fe-Zr-B-based metallic glasses.

Based on the above results, we designed an amorphous composite with a flattened (−Δ*S_m_*)-*T* curve by using 44% (wt.%) Fe_87_Ce_8_B_5_ + 3% (wt.%) Fe_87_Ce_7_B_6_ + 53% (wt.%) Fe_87_Ce_6_B_7_ ribbons. The high −∆*S_m_^average^* (~3.29 J/(kg × K)) and *RC_eff_* (~123.4 J/kg) from 282.5 K to 320 K under 5 T indicate that the hybrid is a promising candidate as a refrigerant applied in a domestic magnetic refrigeration appliance.

## Figures and Tables

**Table 1:** The thermal properties and magnetic properties of the Fe_87_Ce_13−x_B_x_ (x = 5, 6, 7) amorphous ribbons.

Composition	*T_g_* (K)	*T_x_* (K)	*T_l_* (K)	*T_rg_*	*γ*	*T_c_* (K)
x = 5	651	741	1442	0.451	0.354	283
x = 6	698	767	1443	0.483	0.358	305
x = 7	701	777	1444	0.485	0.362	323
	−Δ*S_m_^peak^* (J/(kg × K))					
	1 T	1.5 T	2 T	3 T	4 T	5 T
x = 5	1.08	1.48	1.84	2.49	3.09	3.66
x = 6	1.14	1.56	1.94	2.63	3.28	3.88
x = 7	1.12	1.52	1.89	2.56	3.17	3.75

## Data Availability

Data sharing is not applicable.
